# Supplementary material for: Utilizing co-abundances of antimicrobial resistance genes to identify potential co-selection in the resistome
Source: Microbiol Spectr. 2024 Jun 4;12(7):e04108-23. doi: 10.1128/spectrum.04108-23 (PMC11218503; doi:10.1128/spectrum.04108-23)
Supplement: Supplementary material — Table S1; Fig. S1 to S8. [file spectrum.04108-23-s0001.pdf]

Supplementary materials

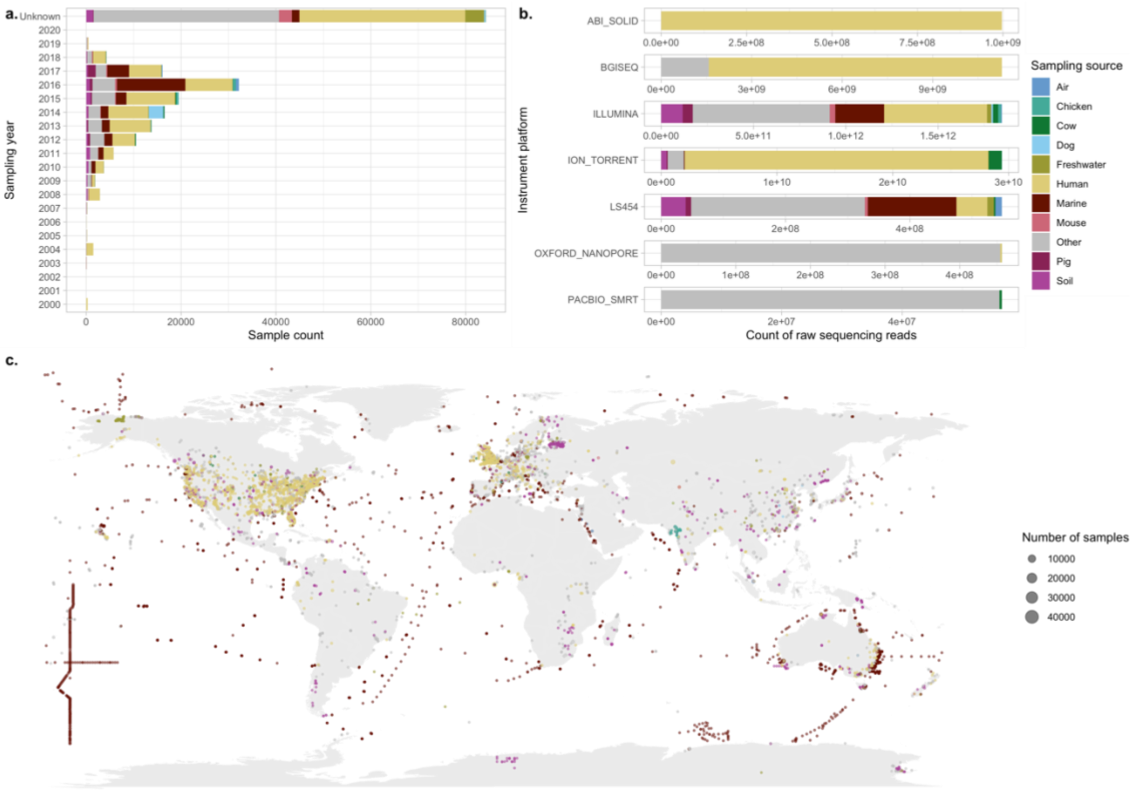

Figure S1: ***Metagenomic origins colored by the sampling group****.* ***a****. Overview of sampling year for metagenomic samples colored by their sampling origin. 100 samples were taken before 2000, and 84,238 did not have a valid sampling date.* ***b****. Overview of the amount of sequencing reads available for each sampling origin.* ***c****. The sampling locations of metagenomic samples used in the correlation analysis were split by their sampling source. Number of samples with no coordinates available; All: 83,361; Air: 16; Dog: 3,159; Chicken: 570; Cow: 262; Freshwater: 61; Human: 40,003; Marine: 363; Mouse: 2,842; Pig: 320; Soil: 477. The 'Other' label refers to those that are not in one of the source-specific networks but are included in the 'All' network.*

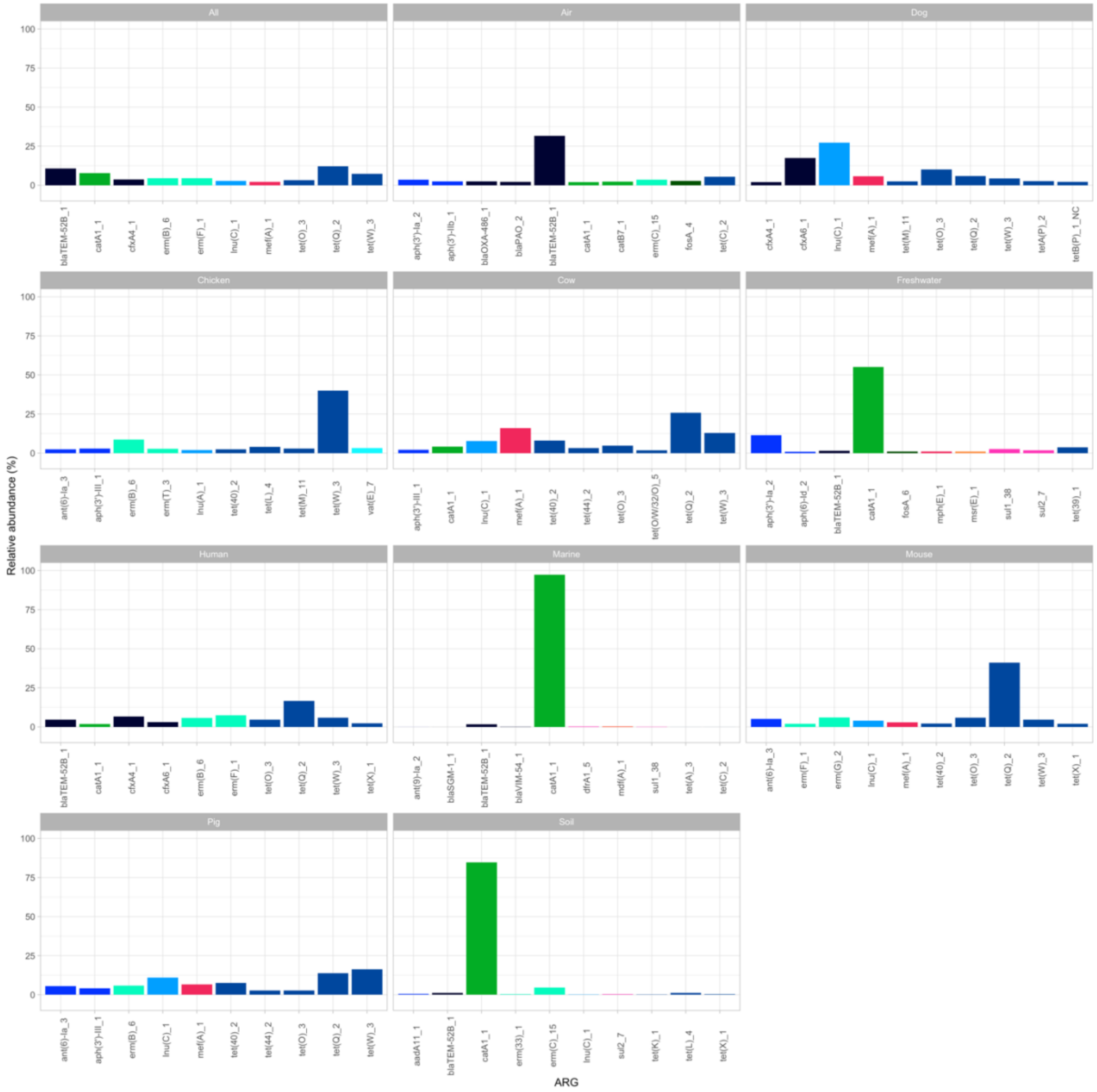

Figure S2: ***Top 10 most abundant ARGs per sampling group.*** *A barplot showing the ARGs having the highest 10 relative abundances per sampling source colored by resistance class.*

	Filter		Network		
Source	minF	minS	I	N	E
All	0	1	716	447	35,573
	25	10	459	241	3,899
	50	10	425	230	2,393
	50	20	313	137	223
	50	50	313	137	223
Air	0	1	436	314	15,079
	25	10	34	14	9
	50	10	19	9	5
Dog	0	1	158	113	1,318
	25	10	45	19	24
	50	10	38	14	17
	50	20	19	7	4
	50	50	19	7	4
Chicken	0	1	389	323	15,658
	25	10	145	114	235
	50	10	123	93	179
	50	20	69	52	71
	50	50	69	52	71
Cow	0	1	443	365	25,632
	25	10	94	72	179
	50	10	85	63	141
	50	20	37	23	32
	50	50	37	23	32
Freshwater	0	1	579	474	19,645
	25	10	56	32	57
	50	10	36	18	23
Human	0	1	692	462	48,821
	25	10	307	158	1210
	50	10	278	141	774
	50	20	201	75	86
	50	50	201	75	86
Marine	0	1	668	15	9
	25	10	24	5	4
	50	10	10	5	4
Mouse	0	1	326	253	10,411
	25	10	55	38	100
	50	10	41	34	92
	50	20	25	21	60
	50	50	25	21	60
Pig	0	1	433	383	21,488
	25	10	136	109	743
	50	10	118	96	526

	Filter		Network		
Source	minF	minS	I	N	E
	50	20	61	49	141
	50	50	61	49	141
Soil	0	1	613	433	9,046
	25	10	72	43	57
	50	10	47	22	22

Table S1: Count filtering of the observed ARG counts (I) greatly influences the number of ARGs (nodes, N) and the correlations (edges, E). For an ARG to be included in the SparCC analysis, it had to pass the two following criteria: it had to have a minimum number of fragments detected (minF) and had to pass the minF criteria for a minimum number of samples (minS).

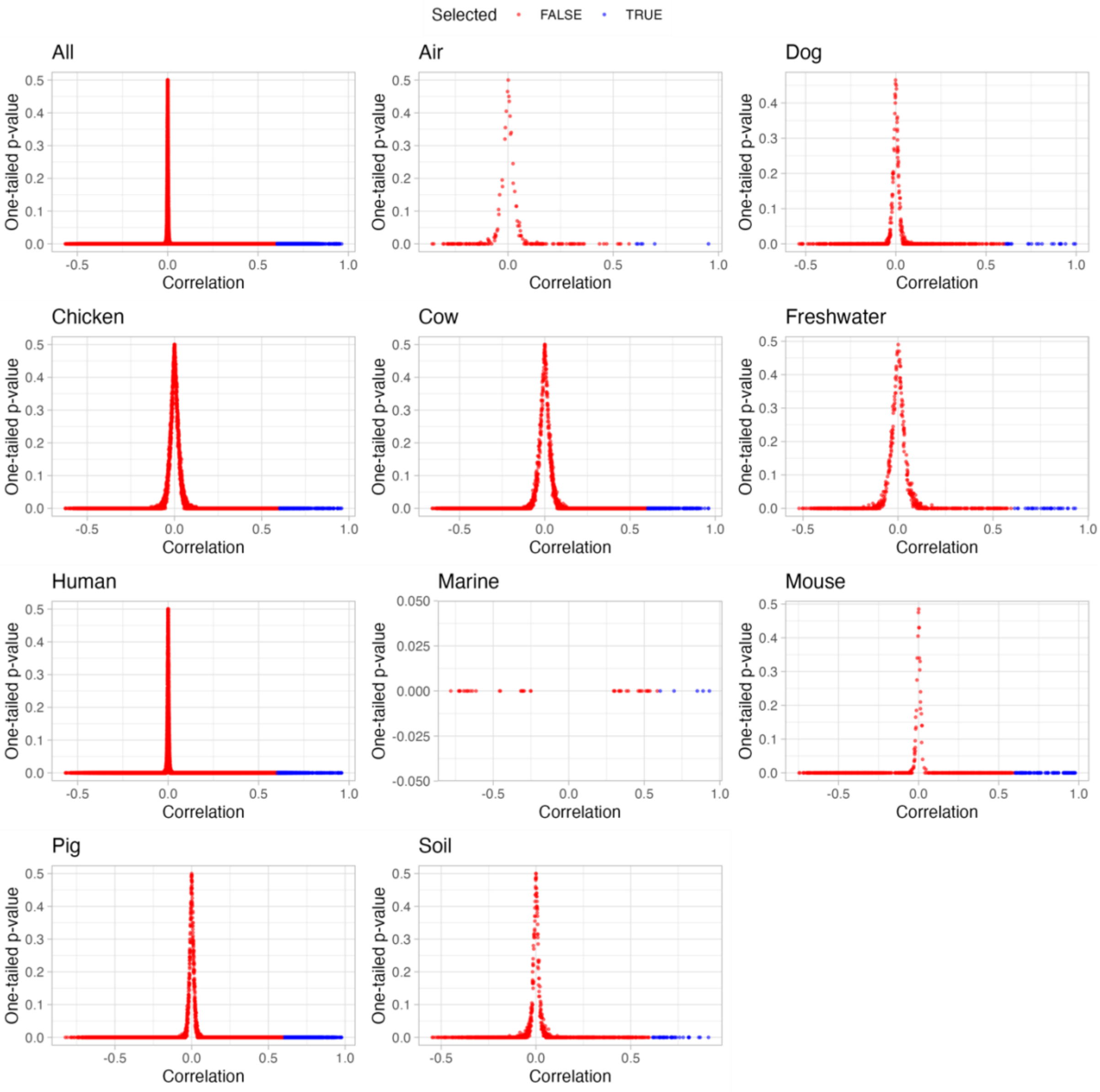

Figure S3: ***Distribution of correlations and p-values produced by SparCC for each data grouping****. Each point is a correlation between two ARGs and is colored by whether the point was selected if the p-value < 0.01 and correlation ≥ 0.6.*

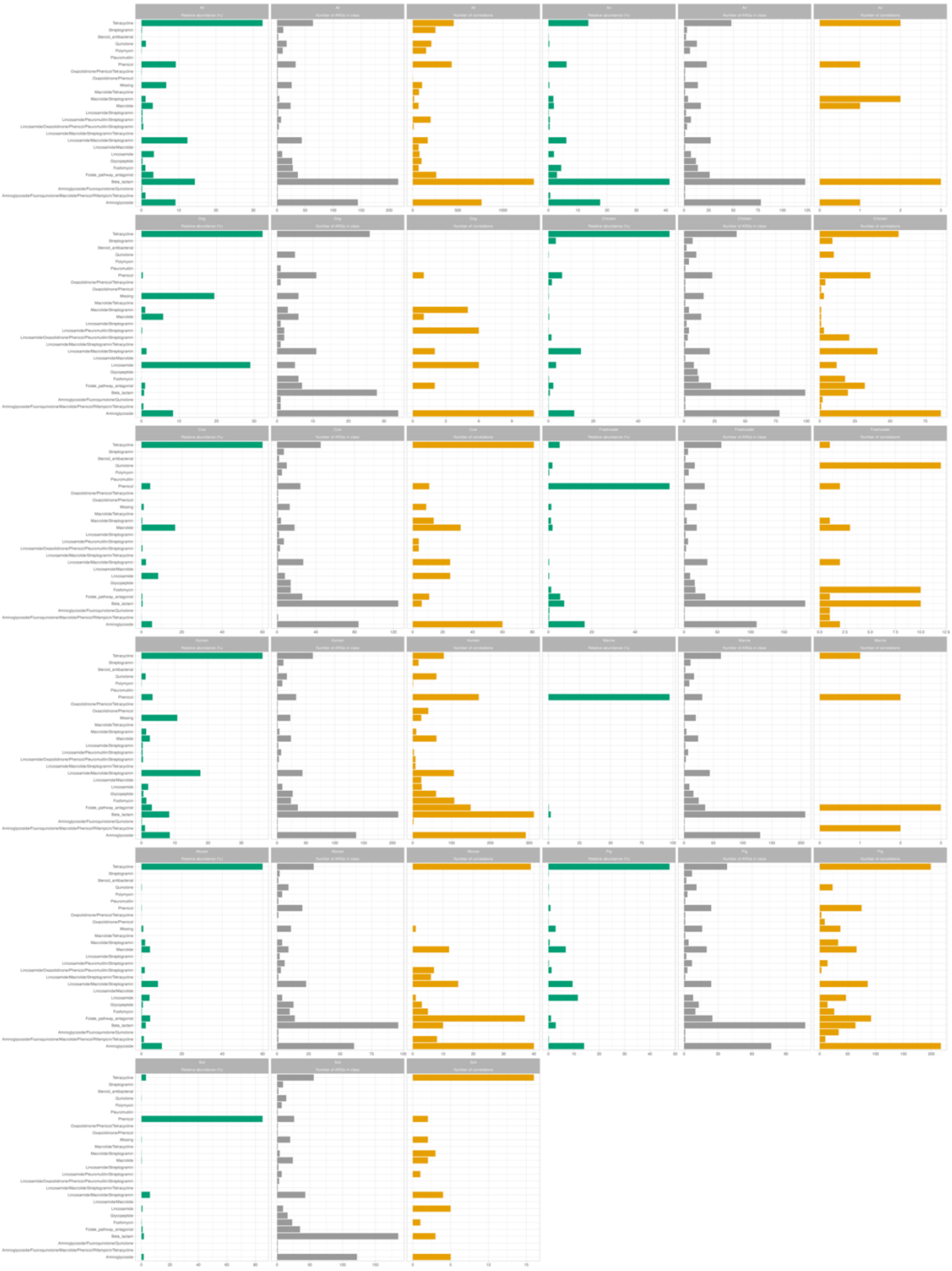

Figure S4: ***The relative abundance, number of ARGs, and the number of correlations for each resistance class in each sampling group.*** *The relative abundance in green shows the percentage of read fragments for each class. In grey is the number of ARGs for each resistance class. Orange coloring indicates the number of correlation coefficients inferred for ARGs in a resistance class.*

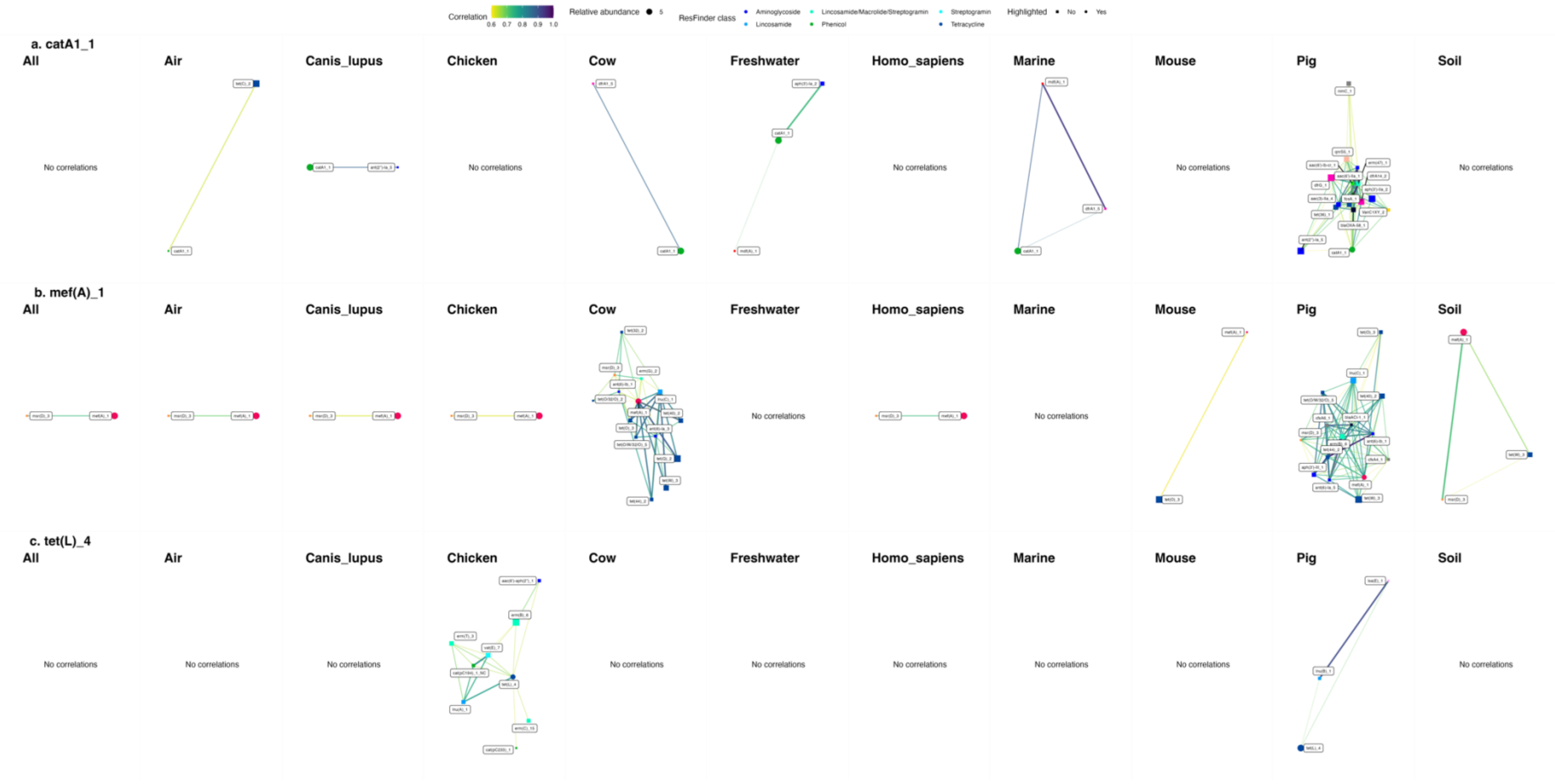

Figure S5: ***Networks illustrating how one ARG interacts differently depending on the sampling environments****. The three genes catA1_1, mef(A)_1, and tet(L)_4 are selected to illustrate that the genes correlate more or less with other genes in their abundance in some habitats.*

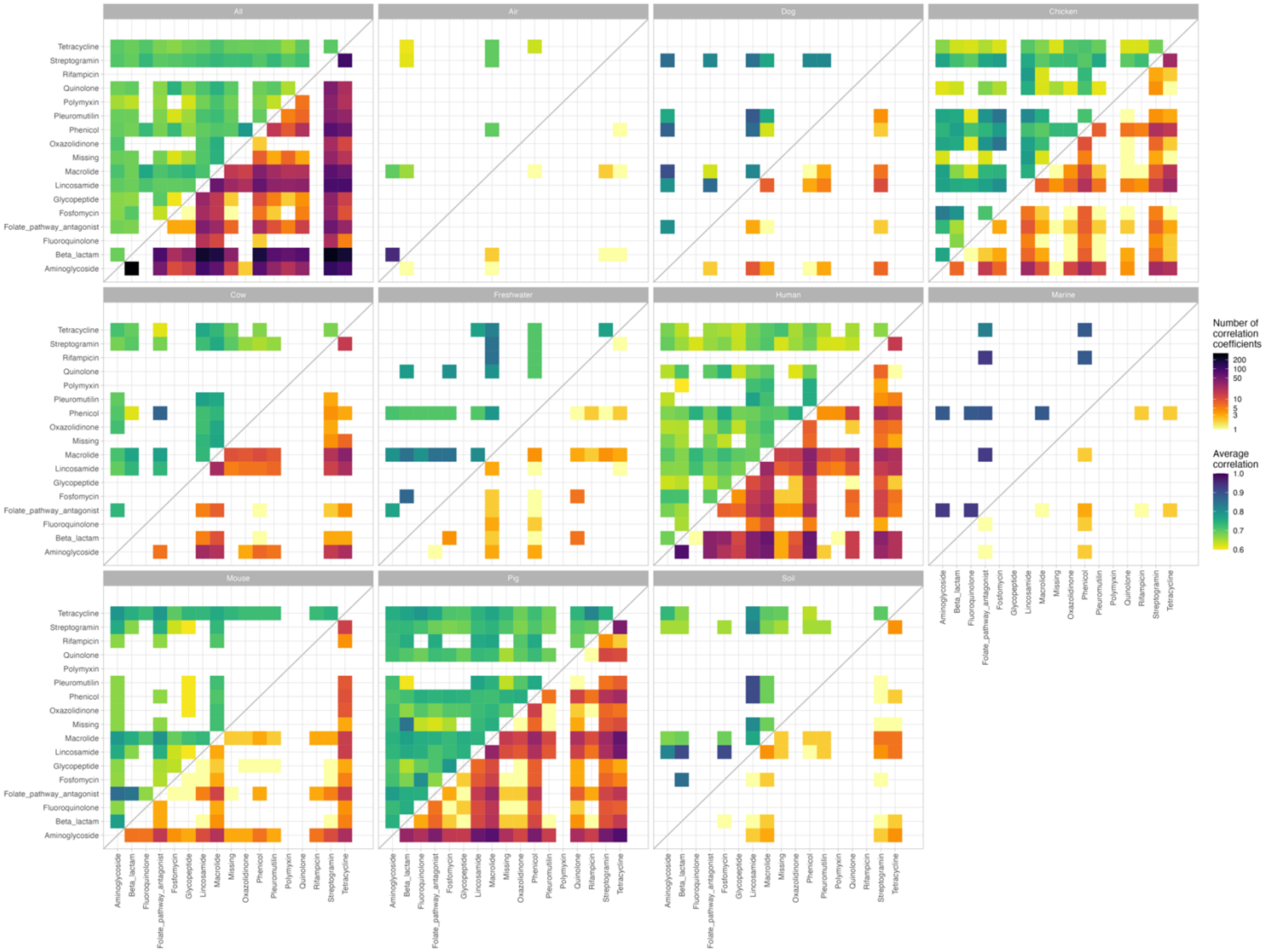

Figure S6: The average correlation between ARGs of different resistance classes in the upper triangle of the heatmaps with the lower half shows the correlation coefficients between the two classes. Note that a correlation coefficient between two ARGs might be present in more than one tile, as some ARGs confer resistance to multiple classes of antimicrobials.

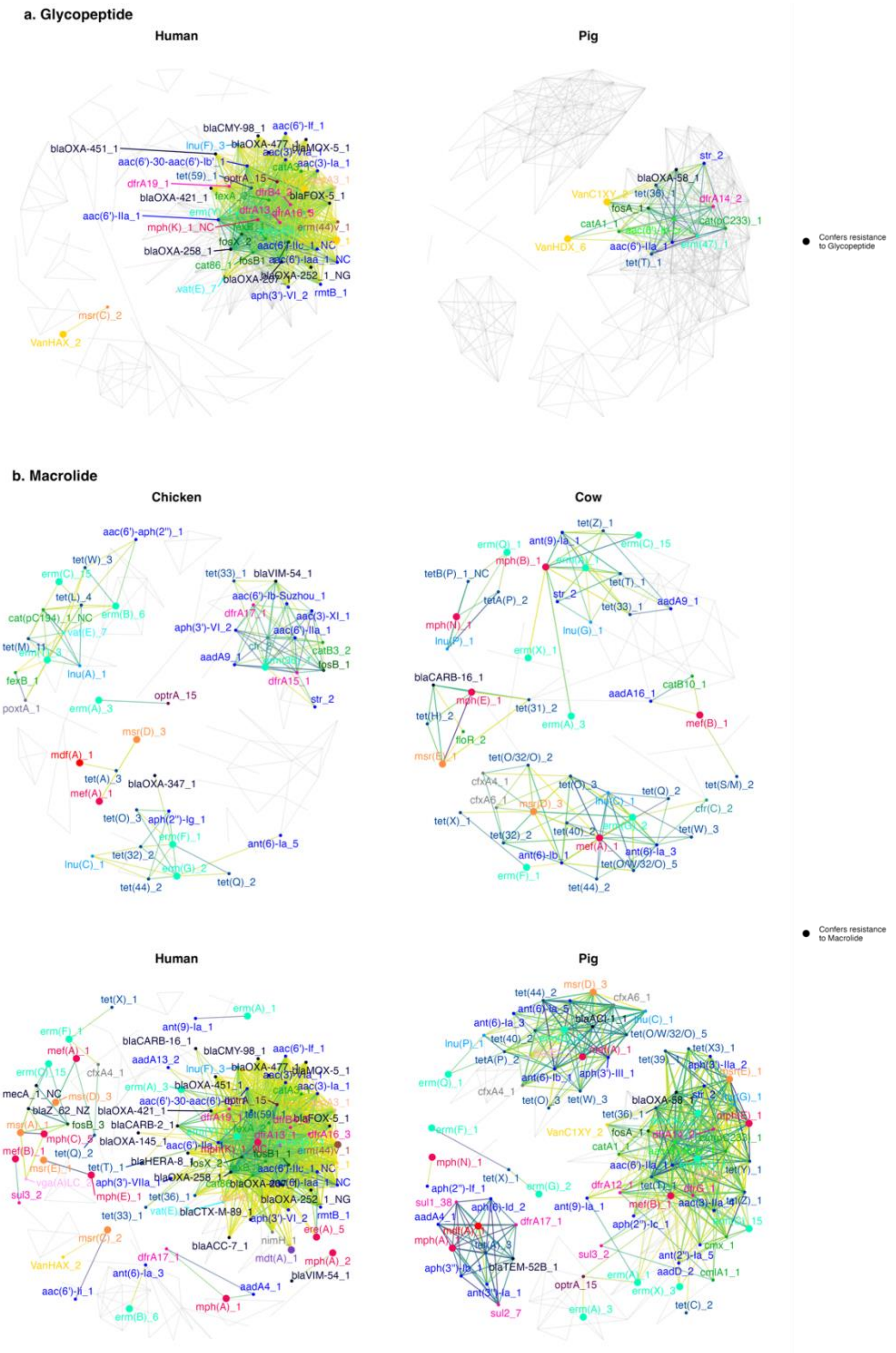

*Figure S7: **Highlights of interactions between ARGs of a. glycopeptide resistance and b. macrolide resistance**. The ARG node size shows whether the corresponding ARG gives resistance to the antimicrobial class in focus. Only correlation edges and ARG nodes are colored if they correlate with the highlighted ARGs; otherwise, they are colored grey. The coloring schemes for nodes and edges are given in Figure 1a.*

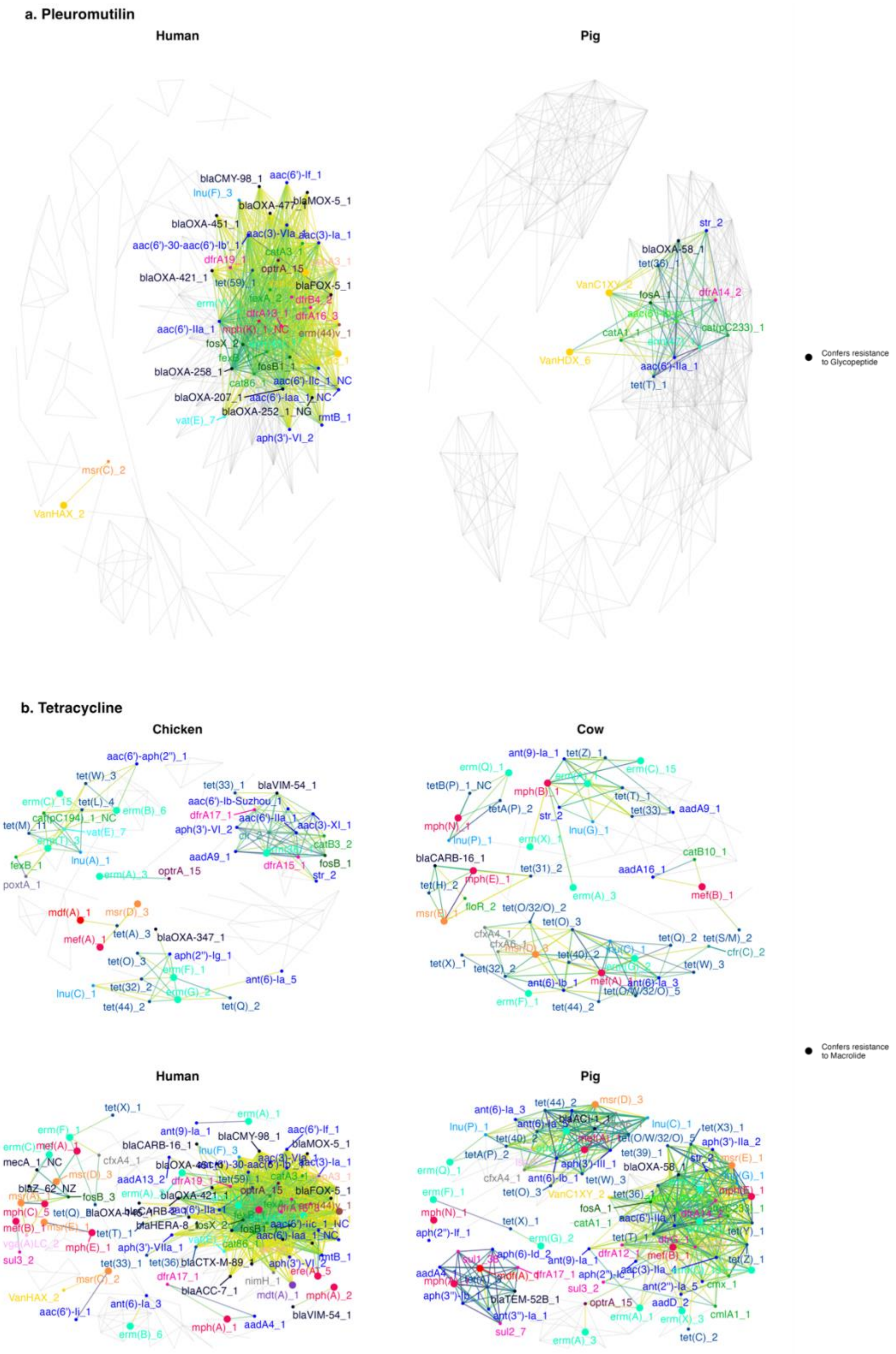

Figure S8: ***Highlights of interactions between ARGs of a. pleuromutilin resistance and b. tetracycline resistance.*** *The ARG node size shows whether the corresponding ARG gives resistance to the antimicrobial class in focus. Only correlation edges and ARG nodes are colored if they correlate with the highlighted ARGs; otherwise, they are colored grey. The coloring schemes for nodes and edges are given in Figure 1a.*
